# Designing for data sharing: Considerations for advancing health equity in data management and dissemination

**DOI:** 10.1093/tbm/ibae049

**Published:** 2024-09-27

**Authors:** Borsika A Rabin, Justin D Smith, Emily V Dressler, Deborah J Cohen, Rebekka M Lee, Melody S Goodman, Heather D’Angelo, Wynne E Norton, April Y Oh

**Affiliations:** Herbert Wertheim School of Public Health and Human Longevity Science, University of California San Diego, La Jolla, CA, USA; UC San Diego Altman Clinical and Translational Research Institute Dissemination and Implementation Science Center, University of California San Diego, La Jolla, CA, USA; Department of Population Health Sciences, Spencer Fox Eccles School of Medicine, University of Utah, Salt Lake City, UT, USA; Utah Clinical and Translational Sciences Institute, Spencer Fox Eccles School of Medicine, University of Utah, Salt Lake City, UT, USA; Division of Public Health Sciences, Department of Biostatistics and Data Science, School of Medicine, Wake Forest University, Winston-Salem, NC, USA; Department of Family Medicine, Oregon Health & Science University, Portland, OR, USA; Harvard T.H. Chan School of Public Health, Harvard University, Boston, MA, USA; School of Global Public Health, New York University, New York, NY, USA; Division of Cancer Control and Population Sciences, National Cancer Institute, Rockville, MD, USA; Division of Cancer Control and Population Sciences, National Cancer Institute, Rockville, MD, USA; Division of Cancer Control and Population Sciences, National Cancer Institute, Rockville, MD, USA

**Keywords:** data sharing, community engagement, NCI, population health, implementation science, health equity

## Abstract

Data sharing, the act of making scientific research data available to others, can accelerate innovation and discoveries, and ultimately enhance public health. The National Cancer Institute Implementation Science Centers in Cancer Control convened a diverse group of research scientists, practitioners, and community partners in three interactive workshops (May–June 2022) to identify and discuss factors that must be considered when designing research for equitable data sharing with a specific emphasis on implementation science and social, behavioral, and population health research. This group identified and operationalized a set of seven key considerations for equitable data sharing—conceptualized as an inclusive process that fairly includes the perspectives and priorities of all partners involved in and impacted by data sharing, with consideration of ethics, history, and benefits—that were integrated into a framework. Key data-sharing components particularly important for health equity included: elevating data sharing into a core research activity, incorporating diverse perspectives, and meaningfully engaging partners in data-sharing decisions throughout the project lifecycle. As the process of data sharing grows in research, it is critical to continue considering the potential positive and adverse impact of data sharing on diverse beneficiaries of health data and research.

## Teaser

Data sharing is a key strategy for advancing our understanding of human health and healthcare. There are opportunities to incorporate health equity into data sharing practices, particularly with populations that are historically underrepresented in research and in partnership with communities plagued by health inequities.

**Implications**

**Researchers:** Researchers need to develop and implement data-sharing plans that incorporate the needs and priorities of all partners and beneficiaries.

**Practitioners:** Practitioners should partner with researchers in shaping the data sharing plan, guiding its implementation, and informing best practices for data sharing.

**Policymakers:** Policymakers should incorporate clear expectations regarding the engagement and incorporation of needs and priorities of all partners and beneficiaries in the data-sharing plan.

## The Potential of Data Sharing

Data sharing, the act of making data from scientific research available to others, has the potential to accelerate the efficient development of equitable population health solutions, particularly in the area of social, behavioral, and implementation sciences [1]. Data sharing is a key strategy for advancing our understanding of human health and healthcare [2]. Despite its potential, implementation of data-sharing activities has been slow and ridden with challenges at multiple levels [3, 4]. Nevertheless, notable successes in data sharing have been realized in genetics [5] and neuroscience [6]. To maximize the benefit of the data collected across single research studies, there is growing recognition of the need to expand data-sharing initiatives more broadly within behavioral and implementation sciences and healthcare research [1, 7–9]. Yet, there are unique complexities when data sharing involves variables and multilevel data types not typically measured in biomedical studies, such as cost and organizational characteristics (e.g. size, culture, and location), individual/patient health-related attitudes and behaviors, and individual data related to health-related attitudes, behaviors, preferences, and priorities. As such, data sharing in this context can be challenging [3] and requires a thoughtful multi-pronged approach [10, 11].

The history and context of communities and past experiences with research have impacted trust, participation, and engagement with research and data [4]. The disciplines of implementation science and public health often consider multiple levels and data types (e.g. policy, community, healthcare system, organizational, and individual). Scientific questions often interact with multiple partners at each level in research and practice. Most importantly, data sharing must reflect a balance between participants’ privacy and the greater good that can come from sharing knowledge more broadly and learning from others.

To address this, the National Cancer Institute Implementation Science Centers in Cancer Control convened a diverse group of scientists, practitioners, and community partners in three interactive virtual workshop sessions, in May–June 2022, to identify factors that would facilitate and barriers that could impede data sharing efforts in implementation science and cancer prevention and control [12]. Interactive, virtual workshops addressed different topical foci, and included a range of experts in data science, ethics, data security and privacy, implementation science, community-engaged research, and cancer prevention and control.

Each session lasted up to 4 h and fostered information sharing and dialogue on the process, scope, and trajectory for data sharing of implementation science data types. A total of 248 attendees participated in these interactive sessions. They reflected practitioner, researcher, funder audiences, and across multidisciplinary expertise: implementation science, data science, epidemiology, behavioral science, community health research and practice, public health, and qualitative, quantitative, and mixed-methods research. For the agenda and recordings from this event, see https://cancercontrol.cancer.gov/sites/default/files/2023-04/35238_DCCPS_IS_Data_Sharing_Workshop_Meeting_Summary_v05_Release_508.pdf. Using lessons from this workshop series, we developed a framework that incorporates key considerations for equitable data sharing. While the focus of the series was data sharing for implementation science, this framework could be broadly applicable to various community-engaged, behavioral, and health systems research. The framework incorporates considerations specific to the new National Institutes of Health (NIH) Data Management and Sharing Policy (DMS) [13], which focuses, in part, on the implication of data sharing on how we interact with community, clinical, and patient/individual research partners.

## Health Equity and Data Sharing

There are opportunities to incorporate health equity into data-sharing practices, particularly with populations that are historically underrepresented in research and in partnership with communities plagued by health inequities. We conceptualized equitable data sharing as a process that considers the perspectives and priorities of all partners involved in and impacted by data sharing. Following community-engaged research principles and practices that facilitate collaborative processes for all phases of research and integrate knowledge and action for mutual benefit for all, researchers can leverage data sharing as a strategy for building trust [14] and communicating findings with community partners with an eye toward creating meaningful and equitable change. Data-sharing practices can also be structured to acknowledge the role of community partners in data collection and ensure transparency and equity data ownership by or with community partners. When data sharing synthesizes research data from multiple communities, it can also enhance the generalizability of findings which is a key facilitator of moving evidence rapidly into action across diverse populations and communities.

## Designing for Data Sharing: What Matters for Equity?

To harness the potential benefits and address the complex and often daunting challenges associated with data sharing, a new paradigm is needed. For data sharing to reach its full potential, a plan for data management and sharing needs to be incorporated from an early stage of the research process (i.e. during the conception of the research plan)—and practices and priorities around data sharing need to be revisited throughout the study. Designing for data sharing involves a set of strategies that promotes the equitable inclusion of all partners in the planning and management of data sharing with the intent to enhance the usability of, engagement with, and access to research data for all. Designing for Data sharing facilitates broader use of research data to inform data-driven population health innovations and solutions that reflect the priorities, preferences, and contexts of beneficiaries to impact population health outcomes.

**Figure 1:**
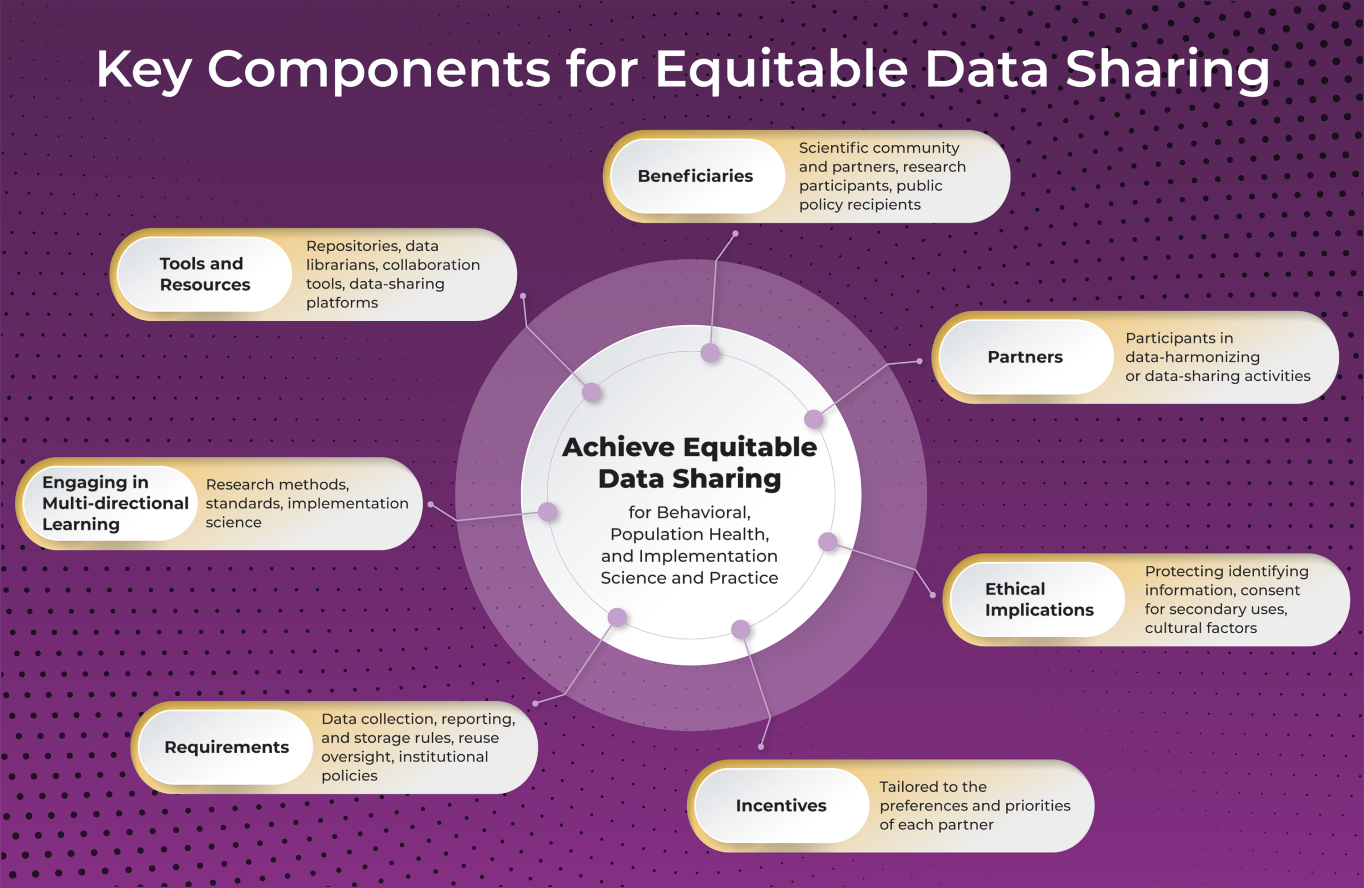
Key components for the equitable data sharing framework

We identify seven key interconnected components for equitable data sharing that are summarized in Fig. 1 and include (i) considerations of the diversity of beneficiaries of data sharing activities, (ii) inclusion of partners, (iii) ethical implications, (iv) identification of incentives, (v) specification of requirements, (vi) multidirectional learning, and (vii) development and use of data sharing tools and resources. Components of the framework might operationalize differently depending on the type of data being collected and shared. For example, qualitative data which is a common and essential way of exploring behavioral and social determinants will require a different type of data management and sharing plan and raise ethical considerations that might not apply in the context of quantitative data [15].

### Element 1: Partners—engaging early and frequently

We define partners as those who are active participants in and contributors to data-sharing activities with varied expertise, power, and interests, including public health and health services researchers, interventionists, data scientists, leaders, managers, and practitioners in clinical and community settings, as well as technology leaders developing software and platforms for data sharing. Partners should be engaged throughout the study, from planning, design, data collection and management, analysis, reporting results, and data sharing; this ensures considerations related to successful and seamless data curation and eventual sharing are met upfront. To further increase complexity, partners and/or their roles might evolve over the course of the study. It is important to highlight the potential role of community partners as they are not often thought of as partners in data sharing but can be influential in terms of data quality and completeness but also vulnerable to the potential adverse impact of data sharing efforts. For example, tribal health agreements exemplify a direct partnership between communities and researchers [16].

### Element 2: Beneficiaries—who they are and how does data sharing benefit them?

Data sharing starts and ends with the beneficiaries—individuals and/or groups who will be impacted, ideally in a positive way, by data sharing efforts. Key questions researchers can ask to identify beneficiaries include: (i) who is impacted by the data collected? (ii) who might be potential users of the data shared? For example, beneficiaries may include patients, community and clinical implementors, researchers, and organizational leaders. Although beneficiaries might not actively engage with the data sharing activities, these activities nonetheless may impact their professional work and/or personal lives in terms of health-related choices and outcomes. It is essential for success to consider all potential beneficiaries and to understand both the value and potential risk of data sharing which will take different forms depending on the beneficiary group. For example, the benefits of data sharing might manifest in the form of available data for scientific inquiry that allows for greater precision by demographic group or geographic area (and national and local policymakers benefit from these more nuanced findings). Meanwhile, operational leaders might see the benefits of data-sharing efforts leading to overall quality improvements and cost savings in their healthcare systems and the public might appreciate the benefit of data-sharing if it contributes to improved shared decision-making processes when solutions are considered for various care options. In addition, benefits and potential adverse outcomes from data sharing for specific beneficiaries should also be clearly defined and considered.

### Element 3: Ethical implications as interrelated and multilevel

As indicated earlier, data sharing involves several beneficiaries and partners who interact in complex ways throughout the research project. For equitable data sharing, it is critical that ethical considerations are explored and co-defined with the consideration of all parties [4]. Multilevel and commonly interrelated ethical implications include the ongoing protection of identifiable information for both beneficiaries and partners (i.e. need for proper deidentification processes) and data ownership (i.e. who owns the data and who is the final decision maker in its use?), and the short and long term use of data. Participation in data sharing, including secondary use of data after the primary study, needs to be discussed with all partners before data collection happens and be part of the informed consent process. Special considerations need to be made for certain study designs, contexts (e.g. small rural communities and Tribal Nations [17, 18]), and data types (i.e. qualitative data, data from mixed-methods studies) being shared. For example, when working with case study designs or in systems where there are few implementors/delivery agents, data can be more easily connected to an individual or organization even when direct identifiers are removed, leading to potential substantial negative impact and adverse outcomes. Effective data-sharing practices could help restore trust in science and consider historical mistrust in health research [2, 19].

### Element 4: Incentives for partners

Data sharing can be a burden on partners, and the expectation of taking on this burden needs to be addressed to ensure success. Sufficient resources need to be allocated for data-sharing efforts that allow for the inclusion of dedicated personnel to undertake data management and sharing activities. Understanding what salient incentives are for each partner is critical [20], and then subsequent clarity and explicit communication regarding incentives should be made available to all partners. Incentives seen as valuable will vary across partners. Researchers might value opportunities for publication, and leaders of clinical and community organizations could be incentivized by incorporating metrics into research surveys that align with key performance metrics, or are high priority for their communities. In the 2023 NIH DMS policy, specifications include a budget for data sharing. This requirement is intended to ensure researchers plan for and budget relevant activities. Budgets may require subcontracts or service agreements with partners that are decided upon at grant submission to ensure success.

### Element 5: Requirements for successful data sharing

Planning for data sharing includes consideration of funder policies (e.g. NIH DMS policies), policies from academic or research institutions (e.g. Institutional Review Board (IRB)), community or local government (e.g. public health department, tribal councils), and organizational policies (e.g. health care systems and school district). Requirements for data sharing can take multiple forms, including considerations for data collection, reporting, storage rules, and reuse oversight. Policies might have an impact on the frequency and amount of data sharing as well as what data can be shared. For successful data sharing, it is critical to discuss and align data-sharing plans with all relevant policies ahead of data curation.

### Element 6: Engaging in multi-directional learning with partners

Information flow about data sharing needs to be multidirectional, iterative, adaptive, and involve all partners. The purpose of learning around data sharing needs to reflect incremental learning similar to those described in learning health systems by relying on ongoing cycles of plan, do, study, and act with the constant engagement of all partners [21]. Similarly, data-sharing practices around common data elements have been part of large initiatives and, while defined centrally, were negotiated based on input from community, clinical, and research partners.

Information exchange and learning will differ depending on the partners involved in the process and will require multiple communication channels and formats that fit best with the needs and priorities of the partners. In an ideal learning process, basic parameters are defined early on with the understanding that everything can be discussed and creatively considered. It is not the responsibility of partners to become experts in the full process of data sharing. Instead, researchers have the responsibility to coordinate unique expertise and contributions from all partners to inform the full process.

### Element 7: Tools and resources to facilitate data sharing

“Designing for data sharing” and related decision-making and learning can increase accessibility and trust [22]. A wide variety of tools and resources can facilitate data sharing. In designing for dissemination of data, identification, and planning for necessary tools and resources will be helpful in identifying resources, team expertise, and budget. These include (i) repositories, data libraries, and platforms that allow for seamless, user-friendly, and safe transfer, secure storage of, and access to shared data; (ii) materials to define and communicate about benefits and potential adverse outcomes of data sharing, including but not limited to communication about benefits by funding agencies written using plain language principles; and (iii) tools to facilitate shared decision-making with partners about how to operationalize requirements around data sharing, including funders and researchers and researchers and their community, clinical, and operational partners. Tools and resources supporting the sharing of biomedical and non-biomedical data exist and can be adapted for the behavioral and implementation sciences and healthcare research contexts [23–25]. Development and dissemination of tools and resources will necessitate training and investment from funding entities in data-sharing activities.

## Next Steps to Maximize the Potential for Data Sharing

For the benefits of data sharing to be fully realized, plans for data sharing need to be incorporated early in the research process and continue throughout the study to ensure alignment with data sharing needs and priorities of all partners. Data also needs to be shared in a timely manner that is consistent with funding agency policies (e.g. time of the first publication or by the end of the grant period for research funded by the NIH). We also need to establish practices and safeguards that support the follow through with what is proposed in the data sharing plan especially when there are no funding agency requirements for data sharing. The concept we describe here as “designing for data sharing” is the sum of research activities and processes to maximize the potential of research data to equitably contribute to population-level impact. Our framework for achieving equitable data sharing for behavioral, population health, and implementation science and practice also provides several recommendations for supporting equitable data sharing, including:

1. Identify beneficiaries of data sharing early on and specify what their key values, incentives, and potential benefits related to data sharing are.
2. Acknowledge potential adverse impacts from data sharing and put safeguards into place to minimize the likelihood of their occurrence.
3. Engage in multidirectional, ongoing learning, and co-creation with partners around incentives, requirements, and needed training, resources, and tools, including how to disseminate findings.
4. Elevate data sharing from an “add-on” activity to an integral and integrated activity of research with its own scientific focus and dedicated resources which can eventually establish the science of data sharing.

Designing for data sharing will require a paradigm and culture shift for researchers and their partners on how data sharing is defined and operationalized, and what resources and tools will be devoted. Early, open, and ongoing collaboration with all relevant partners and beneficiaries will minimize potential problems and maximize public health impact.

## Welfare of Animals

This article does not contain any studies with animals performed by any of the authors.
